# Is Preoperative Adrenal Insufficiency Screening Necessary for Cardiovascular Thoracic Surgery Patients?

**DOI:** 10.3390/medicina59010152

**Published:** 2023-01-12

**Authors:** Worapaka Manosroi, Pichitchai Atthakomol

**Affiliations:** 1 Endocrine and Metabolism Unit, Internal Medicine Department, Faculty of Medicine, Chiang Mai University, Muang Chiang Mai, Chiang Mai 50200, Thailand; 2 Department of Internal Medicine, Faculty of Medicine, Chiang Mai University, Chiang Mai 50200, Thailand; 3 Orthopedics Department, Faculty of Medicine, Chiang Mai University, Muang Chiang Mai, Chiang Mai 50200, Thailand; 4 Center for Clinical Epidemiology and Clinical Statistics, Faculty of Medicine, Chiang Mai University, Chiang Mai 50200, Thailand

**Keywords:** adrenal insufficiency, cardiovascular thoracic surgery, preoperative screening

## Abstract

*Background*: The association between adrenal insufficiency (AI) and the treatment outcomes of cardiothoracic surgery patients has been little reported. The aims of this study were to investigate the incidence of AI and to compare the post-surgical outcomes of patients with perioperatively treated AI and patients with a normal adrenal response. *Methods*: A 1.5-year prospective study was conducted in 98 patients scheduled for cardiothoracic surgery. Patients were categorized as either AI or normal-adrenal-response patients. Those with AI were treated with stress doses of glucocorticoid perioperatively. The post-surgical outcomes of patients with AI and of those with a normal adrenaline response were analyzed using multivariable analysis. *Results*: The overall incidence of AI was 34.7%. There were no statistically significant differences in post-surgical outcomes, including prolonged hospital stay, postoperative infection, prolonged inotropic drug use and relative AI, between the two groups. Only the rate of hyperglycemia requiring insulin infusion was significantly higher in the AI group than in the non-AI group (OR = 14.15, 95% CI = 1.44–138.60, *p* = 0.02). *Conclusions*: The proper diagnosis and management of AI can result in surgical outcomes in AI patients comparable to those of normal-adrenal-response patients. Non-life-threatening hyperglycemia requiring insulin infusion was found only in the AI group.

## 1. Introduction

Adrenal insufficiency (AI) is a potentially lethal disease if left undiagnosed and untreated. Symptoms of AI can be mild or progress to chronic fatigue, muscle weakness, weight loss and abdominal pain. However, untreated acute AI can result in death. AI can be easily treated if recognized early, reducing morbidity and mortality. Due to the body’s increased demand for cortisol during times of stress, e.g., during surgery, critical illness or trauma, the production of cortisol by the adrenal glands increases almost 10-fold in an effort to maintain homeostasis [1]. This rise in the production of cortisol results from increased activation of the hypothalamic–pituitary–adrenal (HPA) axis. In AI patients, the response during a stress episode is impaired and can lead to an adrenal crisis, a life-threatening condition. A report has shown that in surgical patients, adrenocorticotropic hormone (ACTH) and cortisol reach their maximum levels during the early postoperative period [2]. Normally, the cortisol production rate is 10 mg/day, while in major surgery patients, the cortisol production rate rises to 75–150 mg/day [3]. In patients who have undergone coronary artery bypass graft surgery (CABG), serum cortisol levels reach their peak 30 min postoperatively [4].

Recent guidelines recommend that patients with AI who have been scheduled for surgery be administered perioperative glucocorticoids to reduce the risk of adrenal crisis [5]. The dose of glucocorticoids depends on the anticipated surgical stress. If the estimated surgical stress is high, e.g., CABG or esophagectomy, a high dose of glucocorticoids is warranted. If the anticipated surgical stress is estimated to be less than the maintenance dose of glucocorticoids e.g., dental surgery or biopsy, a glucocorticoid stress dose during the perioperative period is not required [6]. However, the evidence regarding this recommendation is still controversial. Multiple reports have suggested that perioperative stress doses of glucocorticoid may not be essential in patients with confirmed AI or those with HPA axis suppression as there was no difference in hypotensive incidence rates between those who received glucocorticoid versus controls [7,8]. Additionally, glucocorticoid administered perioperatively can potentially induce multiple complications, e.g., impaired wound healing, elevated blood glucose and acute psychosis [9,10].

Longer hospital stays and higher re-admission rates have been observed in hospitalized patients with AI compared to non-AI ones [11]. In cardiothoracic surgery, postoperative relative AI can occur, which can result in persistent hemodynamic instability and prolonged vasopressor use [12,13]. In the setting of cardiothoracic surgery, the diagnosis of AI during hospitalization can be challenging due to in-hospital interference factors, e.g., an altered adrenal axis and an increase in cortisol-binding globulin during a critical illness. A diagnosis of AI and proper preoperative treatment can have a positive effect on outcomes. However, most studies of the incidence of AI and outcomes of cardiothoracic surgery have explored this issue during the postoperative period. Very few preoperative evaluations of AI have been reported [14,15]. In this study, we aimed to (1) determine the preoperative incidence of AI and the hospital outcomes of AI patients undergoing elective cardiothoracic surgery and (2) determine whether preoperative AI screening using the ACTH stimulation test and perioperative AI treatment is worthwhile.

## 2. Materials and Methods

A 1.5-year prospective, non-randomized study was conducted during July 2019–Jan 2021. The study was conducted under the Declaration of Helsinki and the protocol was approved by the Faculty of Medicine, Chiang Mai University ethical committee (ethical number: 201/2562). All patients aged above 18 years who were scheduled for elective cardiothoracic surgery, including CABG, valvular heart surgery, aortic aneurysm surgery or atrial septal defect closure, were recruited. Signed informed consent was obtained from all participants included in the study. Patients were excluded if they (1) had a prior diagnosis of AI, (2) had Cushing’s syndrome, (3) had received glucocorticoids within 1 month prior to the operation, (4) required emergency surgery, (5) had nephrotic syndrome or (6) were currently on an oral contraceptive containing estrogen.

Baseline demographic data, as well as underlying history, history of glucocorticoid use and symptoms of AI, were obtained from patients who met the inclusion criteria. Basic biochemical laboratory tests, length of hospital stay, duration of inotropic use, infection complications, insulin use, the occurrence of critical illness-related corticosteroid insufficiency (CIRCI) and surgical outcomes were recorded. Morning serum cortisol (0800 h) was obtained within 1 month prior to surgery. If morning serum cortisol fell into the indeterminate zone (3.1–14.9 µg/dL), a low-dose ACTH stimulation test was performed to diagnose AI. Patients who were diagnosed with AI received a stress dose of glucocorticoid (hydrocortisone 100 mg intravenous (IV)) prior to the operation, followed by a 200 mg IV drip for 24 h, which was then tapered to a maintenance dose of prednisolone of 15 mg/day, which was administered during the perioperative period and continued until discharge. Patients who had a normal adrenal response received standard care as specified by the surgeons.

### 2.1. ACTH Stimulation Test

The low-dose ACTH stimulation test was conducted between 0900 h and 1300 h by well-trained medical nurses. Serum total cortisol level was measured at 20 and 40 min following intravenous administration of 5 μg of ACTH, which was prepared by diluting a 250 μg ampule of ACTH with normal saline. The mixture was then transferred to 1 mL syringes, which were stored at 2–8 °C for no more than 60 days. Serum total cortisol level was determined via the electrochemiluminescence (ECLIA) method using an Elecsys^®^ Cortisol II assay (Roche Diagnostics).

### 2.2. Definitions

AI was defined as either morning serum cortisol < 3 µg/dL or peak serum cortisol after a low-dose ACTH stimulation test of <15 µg/dL [16]. Patients who did not meet the definition of AI were diagnosed as having a normal adrenal response. CIRCI was defined as random serum cortisol of less than 10 μg/dL during hypotension. Prolonged hospital stay was defined as a period of more than 14 days, the median length of hospital stay in our study. Prolonged duration of inotropic support was defined as a period of more than 48 h postoperatively. A history of exogenous glucocorticoid use was defined as prior use (within 3 weeks before surgery) of any form and route of a glucocorticoid with a dose equivalent of >7.5 mg of prednisolone, >30 mg of hydrocortisone or >0.75 mg of dexamethasone for a period of >3 weeks. A history of herbal/traditional medicine use was defined as self-reported use of an herbal/traditional medicine which was suspected of containing glucocorticoids and had not been prescribed by a conventional medical practitioner within 3 weeks prior to surgery and which had resulted in self-reported rapidly resolved symptoms, e.g., increasing appetite or gaining weight.

### 2.3. Statistical Analysis

The data were analyzed using the STATA program version 15.1 (StataCorp, College Station, TX, USA). Statistical significance was set at *p* < 0.05. Counts and percentages are reported for categorical variables. Means ± standard deviation (SD) are reported for continuous variables. For inferential statistics, categorical variables were analyzed using Fisher’s exact test and continuous variables were analyzed using the independent t-test. Logistic regression analysis was conducted for the multivariable analysis of outcomes and is reported as odds ratios (ORs) with 95% confidence intervals (CI). As there had been no previous studies reporting the ORs of the outcomes of interest, the sample size was determined based on reverse power analysis of at least 80%, which can conclude the outcomes.

## 3. Results

Of the 98 patients (46 females, 52 males) who had adrenal insufficiency screening before surgery, 34.70% (34/98) were documented to have AI. The mean age of the patients was 59.06 ± 11.12 years and the most common underlying medical conditions were hypertension (56.7%) followed by dyslipidemia (53.06%). The majority of the patients had previously undergone CABG surgery (45.92%). There was no statistically significant difference in baseline demographic data between the patients with AI and those with a normal adrenal response. The prevalence of steroid or herbal/traditional medicine use showed no difference between the two groups. Baseline biochemical laboratory tests, other than serum cortisol levels, showed no statistically significant differences between the two groups. Morning serum cortisol and serum cortisol after the ACTH stimulation tests were significantly lower in patients with AI than in those with normal adrenal response. The baseline characteristics are as shown in Table 1.

The mean length of hospital stay in the AI group and the normal-adrenal-response group showed no statistically significant difference (13.08 ± 7.59 days and 11.30 ± 5.11 days, *p* = 0.17, respectively). Additionally, there was no statistically significant difference in the duration of inotropic drugs use between patients with AI and those with a normal adrenal response (121.54 ± 30.42 h and 114.60 ± 39.31 h, *p* = 0.85, respectively). Similarly, for incidence of infection, CIRCI and the number of patients who required insulin infusion, there was no statistically significant difference between the two groups.

Based on multivariate analysis after confounder adjustment (age, sex, body mass index (BMI), hypertensive status, diabetes mellitus, creatinine and hemoglobin levels), only the incidence of insulin infusion use was higher in patients with AI than in normal-adrenal-response patients (OR = 14.15, *p* = 0.02). There was no statistically significant difference between groups in the incidence of prolonged hospital stay, postoperative infection, prolonged inotropic drug use or CIRCI. There was only one postoperative death, so the death rate was not incorporated into the multivariable analysis due to the low number of cases. The data are as shown in Table 2.

## 4. Discussion

This study highlights the substantial incidence of AI in preoperative cardiothoracic surgery patients, although patients diagnosed with AI and treated during the perioperative period showed comparable surgical outcomes to those who had normal adrenal response, with the exception of the incidence of insulin infusion in patients diagnosed with AI.

The reported incidence of AI in the general population ranges from 82 to 144 cases/million for primary AI and from 150 to 280 cases/million for secondary AI [17]. In our study, the incidence of AI was 34.70% which is higher than the incidence in the general population. One previous study reported the incidence of AI as being approximately 25% of 45 patients who had undergone elective CABG [18]. Patients’ reported AI symptoms in this study were not significant in the two groups, and most patients reported no symptoms at all. The reason for the unusually high proportion of asymptomatic AI in our cohort was not immediately obvious. One possible hypothesis for the high incidence of AI in this study is that it may relate to the high rate of herbal/traditional medicine use in patients undergoing surgery. In Thailand, almost half the patients with AI reported a history of unprescribed over-the-counter herbal/traditional medicine use [19]. Studies have documented that some herbal/traditional medicines have been intentionally adulterated with glucocorticoids [20,21]. Some Thai patients believe that these products are safe as they are prepared from natural materials and have been used by humans for many generations. The prolonged use of these substances can cause HPA axis suppression and lead to the occurrence of AI. In this study, there was no significant difference in the number of patients who used herbal/traditional substances or glucocorticoid between the group with AI and the non-AI group; however, the study did not investigate which of the herbal/traditional medicines had been adulterated with glucocorticoid. Further study of this issue is needed. Another possibility is that there was a report that stated that a high incidence of AI was frequently observed in myocardial infarction patients, which is the population included in this cohort [22].

HPA axis stimulation with increased cortisol production is essential in maintaining homeostasis during critical illness and severe stress, e.g., a major operation or life-threatening injury. The rise in the level of this hormone is crucial for sustaining vascular contraction, endothelial function and volume distribution. In addition, cortisol can enhance the sensitivity of catecholamine receptors and can maintain the pressor effect. Meta-analyses of small RCTs have reported that glucocorticoid prophylaxis in all patients with cardiothoracic surgery can lessen the atrial fibrillation rate, reduce the duration of vasoactive drug infusion and shorten hospital stays. Nevertheless, the potential adverse side effects, such as the incidence of hyperglycemia and duration of ventilation time, are significantly increased following glucocorticoid therapy [23,24]. The routine prophylactic use of stress glucocorticoids in cardiothoracic surgery to prevent complications is not recommended in the current guidelines [25].

AI patients admitted to the hospital with a general medical illness had a higher incidence of prolonged length of hospital stay and higher re-admission rates compared to the matched controls. Nevertheless, the in-hospital mortality rate was not increased among AI patients [11]. In cardiothoracic surgery patients, prolonged length of hospital stay has been documented in patients with postoperative relative AI [12]. Only one study reported an association of preoperative AI with the outcome of cardiothoracic surgery, specifically, an increase in blood loss and a decrease in volume balance [18]. That study also reported no relationship between preoperative AI and time to ventilator weaning, duration of ICU admission, the use of vasopressor drugs or length of hospital stay. However, in that study, the AI patients were not treated with stress doses of glucocorticoid during the perioperative period. In our study, patients with a diagnosis of AI preoperatively and who were appropriately treated during the perioperative period demonstrated no difference in length of hospital stay, postoperative infection, duration of inotropic drug use or incidence of CIRCI compared to the normal patient group. This suggests that the early detection of AI and proper management with glucocorticoid results in outcomes comparable to patients with a normal adrenal response. Determining whether there is a significant benefit of early detection in reducing these undesirable outcomes will require further study with randomized controlled trials in AI patients both treated and untreated with stress doses of glucocorticoids. Nevertheless, as AI can be a lethal condition, patient assignment to the untreated arm may lead to the withholding of tests and treatments, which may generate ethical concerns.

The only unfavorable side effect found in the present study was that after perioperative glucocorticoid therapy, patients with AI developed postoperative hyperglycemia and required insulin infusion. High doses of glucocorticoid have an oppositional effect on insulin, reducing the sensitivity of insulin (i.e., increasing insulin resistance), reducing beta cell mass, and reducing insulin synthesis, which can lead to hyperglycemia. Hyperglycemia has been linked to postoperative infection at the surgical site, as well as pneumonia and bacteremia in cardiothoracic surgery patients [26]. In the present study, however, the analysis found no relationship between the incidence of postoperative infection and hyperglycemia requiring insulin infusion (r = 0.08, a negligible correlation). Taken together, as the adverse effect was minimal and not life-threatening, the benefits of AI screening and treatment outweigh the risks, so screening for AI before cardiothoracic surgery is of value. The screening may need to be conducted in institutions with limited diagnostic resources. Therefore, priority should be given to patients at high risk of having AI, including patients reporting long-term use of glucocorticoids or substances suspected of adulteration with glucocorticoids, and patients with symptoms of AI.

The strengths of the present study are as follows: (1) The diagnosis of AI was performed via dynamic ACTH stimulation tests before surgery. This can reduce confounding factors of serum cortisol level at the time of admission, e.g., stress before and after surgery, the alteration of cortisol-binding globulin, and falsely elevated serum cortisol in cases of chronic kidney disease. (2) The multivariate analysis was adjusted for potentially confounding factors. This helped minimize the factors that could potentially affect the outcomes. (3) The study recruited a large number of participants and the sample size had an adequate backward calculation of power (>80%) to interpret the outcomes. (4) Finally, the present study is novel in that all the participants who had AI were administered stress doses of glucocorticoid. This allowed for demonstration of the direct effect of proper treatment on AI patients before surgery.

We acknowledge some limitations in this study. The actual benefit of glucocorticoid treatment versus non-treatment in patients with preoperative AI was not demonstrated in this study as it was an observational study. The hemodynamic outcomes after cardiothoracic surgery, e.g., cardiac index, fluid balance, pulmonary venous pressure, and central venous pressure, were not documented. A low-dose ACTH stimulation test was employed instead of a high-dose test due to the short supply of ACTH in our country. However, a study demonstrated that low- and high-dose ACTH stimulation tests showed similar cortisol responses in patients with AI [27]. Additionally, 5 g of ACTH was used instead of 1 g, to reduce the error of ACTH adhering to the plastic tube during injection, which can lead to an insufficient cortisol response. The causes of AI were not identified via ACTH level, plasma renin, aldosterone or imaging in this study. Low cortisol-binding globulin was observed in cirrhosis patients, and these patients were not excluded from this study. Nevertheless, in our study, all patients with cirrhosis had a normal adrenal response. Finally, we did not provide in-depth details concerning the characteristics of patients who needed to be screened for AI.

## 5. Conclusions

Preoperative AI is frequently observed in cardiothoracic surgery patients and is usually asymptomatic. AI screening followed by appropriate preoperative management in patients undergoing cardiothoracic surgery may have a beneficial effect in terms of postoperative outcome. The only minor adverse side effect of stress-dose glucocorticoid management in AI patients is hyperglycemia, which necessitates intravenous insulin infusion. In patients where there is a strong suspicion of AI and where diagnostic tests are available, morning serum cortisol followed by dynamic ACTH stimulation tests to diagnose AI should be performed and suitable treatment provided. However, this study is single-center and non-randomized; a further multi-center study using randomized groups of AI patients before surgery is warranted.

## Figures and Tables

**Table 1:** Baseline characteristics.

Characteristics	Adrenal Insufficiency (n = 34)	Normal Adrenal Response (n = 64)	*p*-Value
Baseline demographic data			
Age, mean ± SD (yr)	57.67 ± 12.10	59.79 ± 10.59	0.37
Female, n (%)	16 (47.06)	30 (46.88)	0.98
Body weight, mean ± SD (kgs)	59.88 ± 9.96	60.24 ± 11.96	0.87
BMI, mean ± SD (kg/m^2^)	23.37 ± 3.71	23.70 ± 4.03	0.69
Systolic blood pressure, mean ± SD (mmHg)	119.85 ± 21.94	116.59 ± 24.04	0.51
Diastolic blood pressure, mean ± SD (mmHg)	70.67 ± 11.62	70.89 ± 11.58	0.93
Underlying diseases, n (%) - Hypertension - Diabetes mellitus - Dyslipidemia - Chronic kidney disease - Cirrhosis - Others			
	17 (50.00)	38 (60.32)	0.32
	6 (17.65)	20 (31.25)	0.14
	18 (52.94)	34 (53.13)	0.98
	5 (14.71)	8 (12.50)	0.75
	0 (0)	3 (4.69)	0.20
	17 (50.00)	29 (42.65)	0.48
Type of cardiothoracic surgery, n (%) - CABG - Valvular heart surgery - Aortic aneurysm surgery - ASD closure			
	11 (32.35)	34 (53.13)	
	19 (55.88)	26 (40.63)	
	1 (2.94)	0 (0)	
	3 (8.82)	4 (6.25)	0.15
Symptoms of adrenal insufficiency, n (%) - Nausea/vomiting - Significant weight loss - Others - None			
	1 (2.94)	2 (3.13)	
	6 (17.65)	10 (15.63)	
	6 (17.65)	3 (4.69)	
	21 (61.76)	49 (76.56)	0.18
History of exogenous glucocorticoid use, n (%)	1 (2.94)	5 (7.81)	0.33
History of herbal/traditional medicine use, n (%)	11 (32.35)	15 (23.44)	0.34
Baseline biochemical investigations			
8AM Serum cortisol, mean ± SD (µg/dL)	8.30 ± 2.97	10.50 ± 3.26	<0.005
ACTH stimulation test - 0 min cortisol, mean ± SD (µg/dL) - 20 min cortisol, mean ± SD (µg/dL) - 40 min cortisol, mean ± SD (µg/dL)			
	6.88 ± 2.46	10.12 ± 3.29	<0.005
	13.50 ± 1.94	18.71 ± 2.96	<0.005
	14.97 ± 2.28	21.86 ± 2.97	<0.005
Serum albumin, mean ± SD (g/dL)	4.07 ± 0.49	4.15 ± 0.47	0.38
Hemoglobin level, mean ± SD (g/dL)	12.60 ± 2.10	12.25 ± 1.84	0.39
Total cholesterol, mean ± SD (mg/dL)	149.91 ± 42.48	151.21 ± 40.06	0.88
LDL, mean ± SD (mg/dL)	98.5 ± 37.31	100.38 ± 38.43	0.81
HDL, mean ± SD (mg/dL)	44.52 ± 14.06	44.28 ± 15.36	0.93
Creatinine, mean ± SD (mg/dL)	1.13 ± 0.61	1.19 ± 1.45	0.82
Serum sodium, mean ± SD (mmol/L)	139.26 ± 2.89	137.07 ± 17.12	0.46
Serum potassium, mean ± SD (mmol/L)	4.06 ± 0.48	4.41 ± 2.91	0.52
Serum bicarbonate, mean ± SD (mmol/L)	27.08 ± 14.13	25.53 ± 9.26	0.51

BMI: body mass index; CABG: coronary artery bypass graft; ASD:atrial septal defect; AM: is the time (I think there is no need to define abbreviation here); ACTH: adrenocorticotropin; LDL: low density lipoprotein; HDL: high density lipoprotein.

**Table 2:** Multivariable analysis of postoperative outcomes and screening for adrenal insufficiency.

Factors	Adrenal Insufficiency (n = 34)	Normal Adrenal Response (n = 64)	Adjusted ORs *	95% Confidence Interval	*p*-Value
Prolonged length of hospital stay (>14 days)	11 (32.32)	16 (25.00)	1.67	0.55–4.99	0.35
Postoperative infection	7 (20.59)	14 (21.88)	1.16	0.38–3.56	0.79
Prolonged inotropic drug use (>48 h)	2 (5.88)	3 (4.69)	1.45	0.20–10.32	0.71
CIRCI	11 (32.35)	19 (29.69)	1.18	0.44–3.15	0.74
Required insulin infusion	8 (23.53)	9 (14.06)	14.15	1.44–138.60	0.02

ORs: odds ratio. * Adjusted for age, sex, body mass index, hypertensive status, diabetes mellitus, creatinine and hemoglobin level. CIRCI: critical illness-related corticosteroid insufficiency.

## Data Availability

The datasets generated and/or analyzed during the current study are not publicly available due ethical reasons but are available from the corresponding author upon reasonable request.

## Abbreviations

AI	Adrenal insufficiency
ACTH	Adrenocorticotropic hormone
CABG	Coronary artery bypass graft
CIRCI	Critical illness-related corticosteroid insufficiency
CI	Confidence interval
HPA	Hypothalamic–pituitary–adrenal axis
IV	Intravenous
OR	Odds ratio
RCT	Randomized controlled trial
SD	Standard deviation
